# Diagnosis and treatment of rotatory knee instability

**DOI:** 10.1186/s40634-019-0217-1

**Published:** 2019-12-21

**Authors:** Jonathan D. Hughes, Thomas Rauer, Christopher M. Gibbs, Volker Musahl

**Affiliations:** 1 Department of Orthopaedic Surgery, UPMC Freddie Fu Sports Medicine Center, University of Pittsburgh, 3200 S. Water St, Pittsburgh, PA 15203 USA; 2 Department of Trauma Surgery, University Hospital Zurich, Zurich, Switzerland

**Keywords:** Rotatory knee instability, Lateral tenodesis, Anterolateral

## Abstract

**Background:**

Rotatory knee instability is an abnormal, complex three-dimensional motion that can involve pathology of the anteromedial, anterolateral, posteromedial, and posterolateral ligaments, bony alignment, and menisci. To understand the abnormal joint kinematics in rotatory knee instability, a review of the anatomical structures and their graded role in maintaining rotational stability, the importance of concomitant pathologies, as well as the different components of the knee rotation motion will be presented.

**Main Body:**

The most common instability pattern, anterolateral rotatory knee instability in an anterior cruciate ligament (ACL)-deficient patient, will be discussed in detail. Although intra-articular ACL reconstruction is the gold standard treatment for ACL injury in physically active patients, in some cases current techniques may fail to restore native knee rotatory stability. The wide range of diagnostic options for rotatory knee instability including manual testing, different imaging modalities, static and dynamic measurement, and navigation is outlined. As numerous techniques of extra-articular tenodesis procedures have been described, performed in conjunction with ACL reconstruction, to restore anterolateral knee rotatory stability, a few of these techniques will be described in detail, and discuss the literature concerning their outcome.

**Conclusion:**

In summary, the essence of reducing anterolateral rotatory knee instability begins and ends with a well-done, anatomic ACL reconstruction, which may be performed with consideration of extra-articular tenodesis in a select group of patients.

## Background

**Fig. 1:**
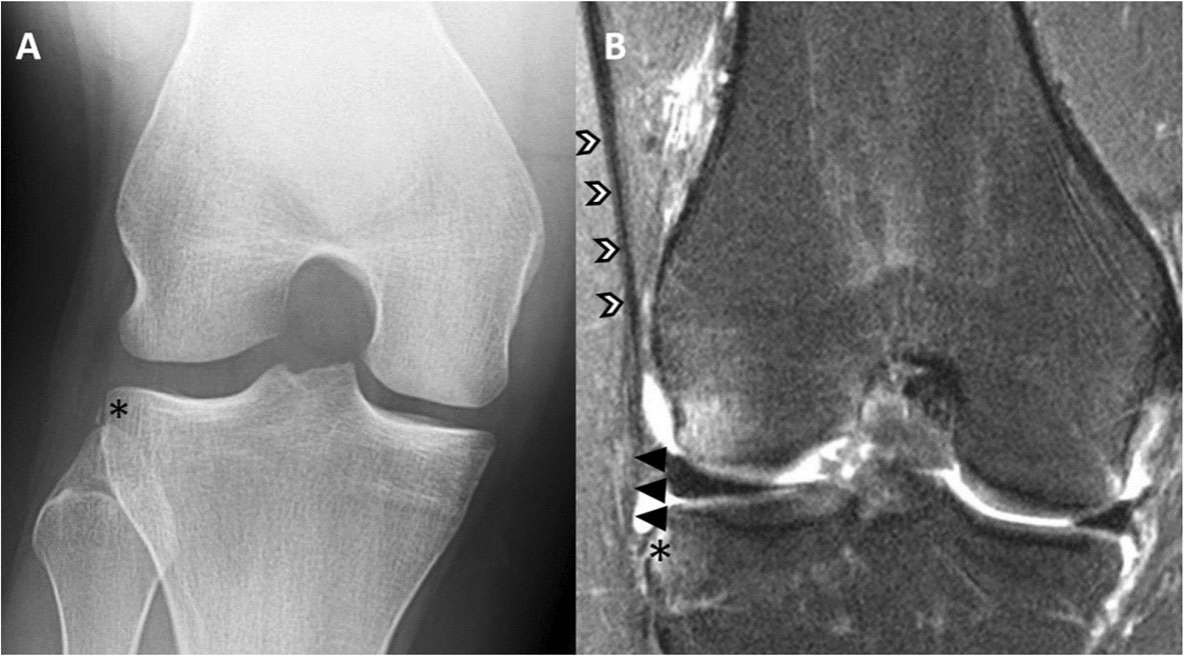
Radiograph (**a**) and magnetic resonance imaging exam (**b**) of a Segond fracture with injury to the anterolateral capsule. The black star denotes the Segond fracture, or an avulsion fracture off the lateral tibial plateau. The black and white errors denote the iliotibial band, while the black arrows demonstrate the anterolateral capsule

The concept of rotatory knee instability was introduced in 1870 by French surgeon Paul Segond while studying the role of rotation in causing knee injuries with hemarthrosis [63]. Segond’s lone orthopaedic publication provided the first description of knee injuries resulting from forced rotational motion, and earned him the still-used eponym, the Segond Fracture [63] (Fig. 1). In 1968, Slocum described anteromedial rotatory instability after knee injury as a “pathologically increased outward rotation of the tibia on the femur” [88]. In 1976, Hughston et al. introduced a classification system which included anteromedial instability, anterolateral instability, posterolateral instability, or combined rotational injury of the knee [36, 37]. As such, rotatory knee instability is a large and complex topic. Studying this injury in smaller subdivisions of pathology involving the anteromedial, anterolateral, posteromedial, and posterolateral ligaments, bony alignment, and menisci allows one to form an understanding of this vast topic, which is vital to properly diagnose and treat rotatory knee disorders [66]. This report will discuss the etiology, diagnosis, and treatment of each type of rotatory knee instability; however, particular attention will be given to anterolateral rotatory knee instability due to the limited understanding and controversial treatment recommendations surrounding this particular instability pattern and the anterolateral capsular complex.

## Review

### Anteromedial rotatory instability

#### Etiology

Anteromedial rotatory instability (AMRI) results from excessive valgus strain with simultaneous external rotation of the knee, leading to pathologic anterior subluxation of the medial tibial plateau relative to the medial femoral condyle [19]. AMRI can be caused by injury to the superficial and deep medial collateral ligaments (MCL), posterior capsule, and posterior medial corner (PMC). The PMC, which is comprised of the posterior horn of the medial meniscus, posterior oblique ligament (POL), semimembranosus expansions, meniscotibial ligaments, and oblique popliteal ligament, normally functions to provide static and dynamic stabilization to the medial aspect of the knee [86, 88]. Of these various structures, the POL is consistently implicated in different injury patterns [92].

**Fig. 2:**
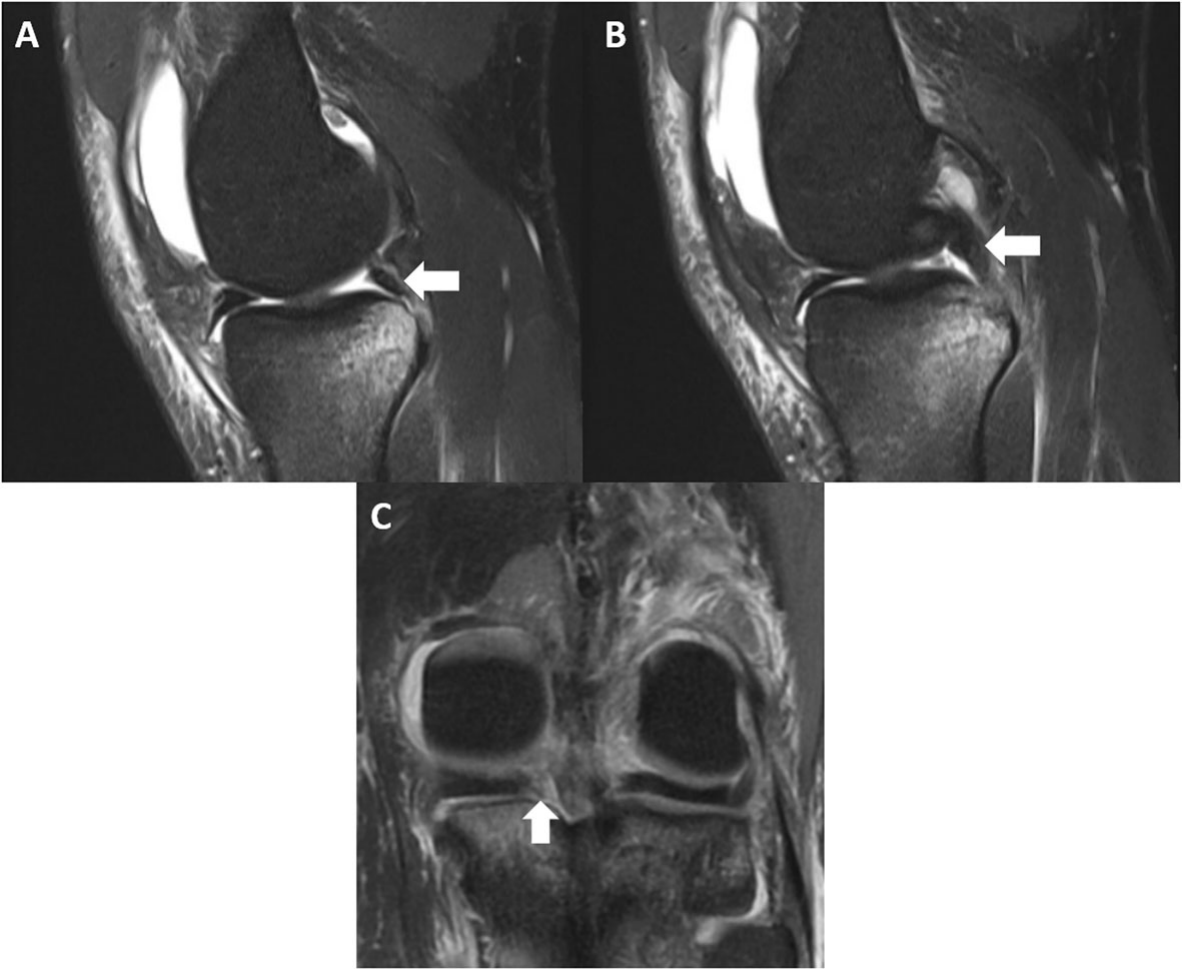
A magnetic resonance imaging of a medial meniscus root tear in conjunction with an anterior cruciate ligament tear. The white arrows point to the meniscus root as it enters its insertion on the tibia. On the top images (**a** and **b**), there is fluid underneath the root with no clear attachment to the tibia. The bottom image (**c**) demonstrates no clear attachment of the root to the tibia

**Fig. 3:**
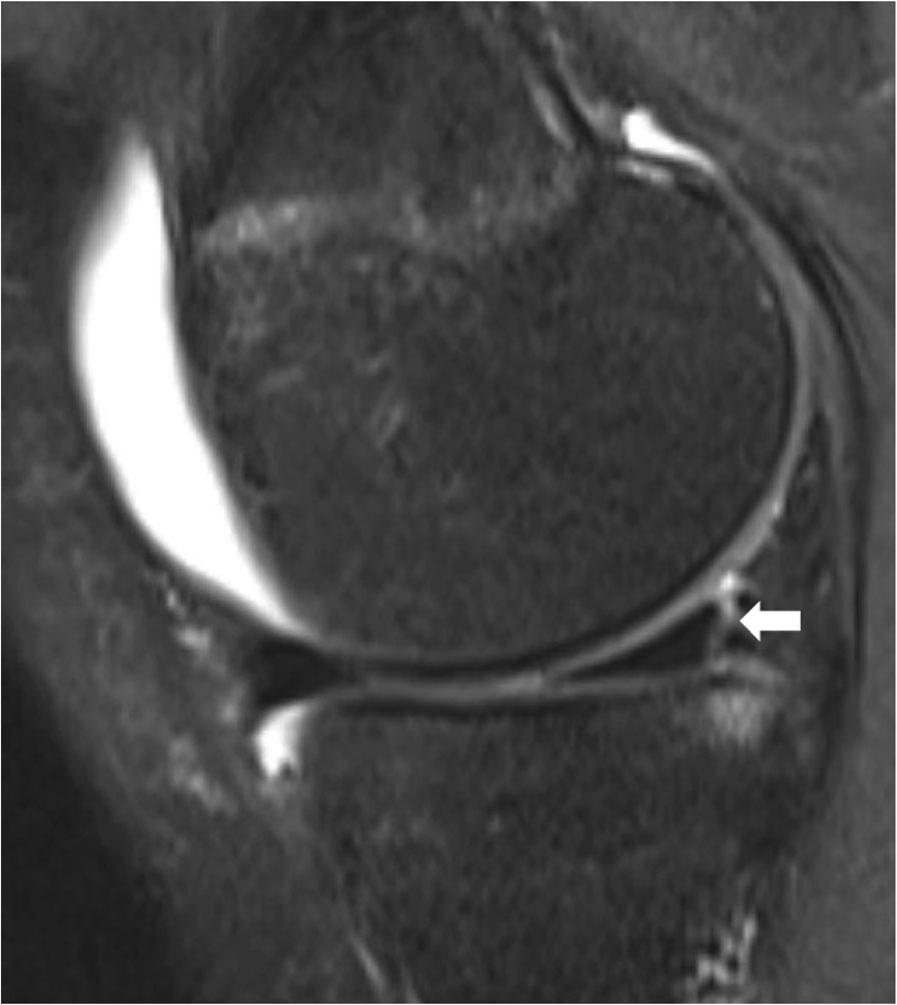
A ramp lesion on a sagittal magnetic resonance imaging exam (MRI). The white arrow points to a peripheral tear in the posterior horn of the medial meniscus, aka a ramp lesion. These lesions are often missed on MRI, and even during arthroscopic examination

Recently, lesions within the posterior horn of the medial meniscus, including root tears (Fig. 2) and ramp lesions, have been implicated in rotatory knee instability [67, 75, 85, 89]. A ramp lesion is a complete, longitudinal lesion of the posterior horn medial meniscus that occurs within the periphery of the meniscus (Fig. 3). These lesions are frequently missed on radiographic examination, and can be missed on arthroscopic examination. A supplemental portal through the notch during arthroscopic examination may be required to fully evaluate for these lesions.

#### Diagnosis

AMRI is clinically evaluated with physical examination findings of pain in the medial and posteromedial aspects of the knee as well as increased laxity with abduction stress applied at 30 degrees of knee flexion, coupled with anterior rotatory subluxation of the medial tibial plateau relative to the corresponding femoral condyle [86]. Another useful clinical test includes the rotatory instability test, which is an anterior drawer test with the knee in 15 degrees of external rotation. Pathologically increased anterior and lateral displacement of the tibia is considered a positive test [88]. A positive dial test, consisting of increased external rotation at 30 and 90 degrees of knee flexion with anterior subluxation of the medial tibial plateau, is also indicative of a complete injury to the medial structures causing AMRI [19]. Finally, meniscal instability manifesting as medial or lateral subluxation can also be assessed during abduction and adduction stress to the knee [86].

Imaging studies can prove useful in the diagnosis of AMRI. Radiographic analysis can reveal increased medial compartment gapping under valgus stress [46]. MRI is also useful for delineating the extent of involvement of medial structures [86, 92].

#### Treatment

Isolated grade I and II MCL injuries are treated nonoperatively, consisting of rest, ice, and elevation with or without bracing and rehabilitation [19, 92]. Isolated treatment can also be considered for grade III MCL injury; however concomitant cruciate ligament injury, or avulsion of the distal superficial MCL external to the pes anserinus insertion, is typically considered an indication for surgical repair of the MCL with or without the POL [19]. A variety of surgical techniques have been described. Acute repair typically involves addressing the superficial or deep MCL and using this for attachment of PMC structures as a sheet of tissue; chronic reconstruction involves various methods of using auto- or allograft to recreate the medial structures of the knee [92].

### Posterolateral rotatory instability

#### Etiology

Posterolateral Rotatory Instability (PLRI) is a relatively rare injury involving injury to the arcuate ligament complex, comprised of the lateral collateral ligament (LCL), arcuate ligament, popliteus muscle and tendon, and lateral head of the gastrocnemius. PLRI usually is the result of a hyperextension, varus moment, and rotatory force on the knee [13]. This causes the lateral tibial plateau to subluxate posteriorly in relation to the lateral femoral condyle [23].

#### Diagnosis

PLRI can be diagnosed on physical exam by multiple exam maneuvers. While ambulating into the examination room, the patient may demonstrate a varus thrust and stance phase knee hyperextension during gait [13]. Increased posterior tibial translation at 30 degrees of knee flexion is indicative of an isolated posterolateral corner (PLC) injury, while increased laxity at 30 and 90 degrees of knee flexion is indicative of concomitant PLC and PCL injury [13]. Lateral compartment widening under varus stress applied with gentle internal rotation of the tibia at 0 and 30 degrees of knee flexion occurs in combined LCL and PLC injuries [13]. The external-rotation recurvatum test is performed by grasping bilateral great toes and lifting the leg off of the examination surface, with positive findings including knee recurvatum, tibial external rotation, and increased varus deformity [13]. The posterolateral external rotation test is performed at both 30 and 90 degrees of knee flexion by applying a posterior force couple with external rotation of the tibia; positive test consisting of posterolateral subluxation of the lateral tibial plateau at 30 degrees only is indicative of isolated PLRI. If posterolateral subluxation of the lateral tibial plateau occurs at both 30 and 90 degrees, concomitant PCL injury should be suspected [13]. A reverse pivot shift test consists of applying a valgus load with the tibia in external rotation while bringing the knee from flexion to extension. A positive test includes a palpable shift or jerk as the posteriorly subluxated medial tibia reduces, indicating possible PLRI [13]. Finally, a standing apprehension test, in which the knee is slightly flexed while bearing weight, is reported to be 100% for PLRI, with a positive test of internal rotation of the lateral femoral condyle relative to the tibial plateau with the subjective feeling of “giving way”.

Imaging studies, including varus stress and full-length weightbearing radiographs, to assess overall limb alignment can be helpful in the diagnosis and surgical planning of PLRI. MRI may also be useful for delineation of injury to individual structures in the PLC [13].

#### Treatment

Cases of mild instability may be managed nonoperatively with a brief period of immobilization followed by rehabilitation in a select group of patients; however, symptomatic instability with functional limitations or PLRI with concomitant cruciate ligament injury necessitates surgical intervention for optimal outcomes [13]. In general, direct repair is preferred and is often possible within 2 weeks of injury; however, in cases in which the tissue quality is not amenable to direct repair or the injury is more chronic in nature, reconstruction with allograft and capsular advancement may be required [23]. Different techniques have been described, including those that are purely fibular-based or those that involved both the tibia and fibula [17, 47, 48].

### Anterolateral rotatory instability

#### Etiology

**Fig. 4:**
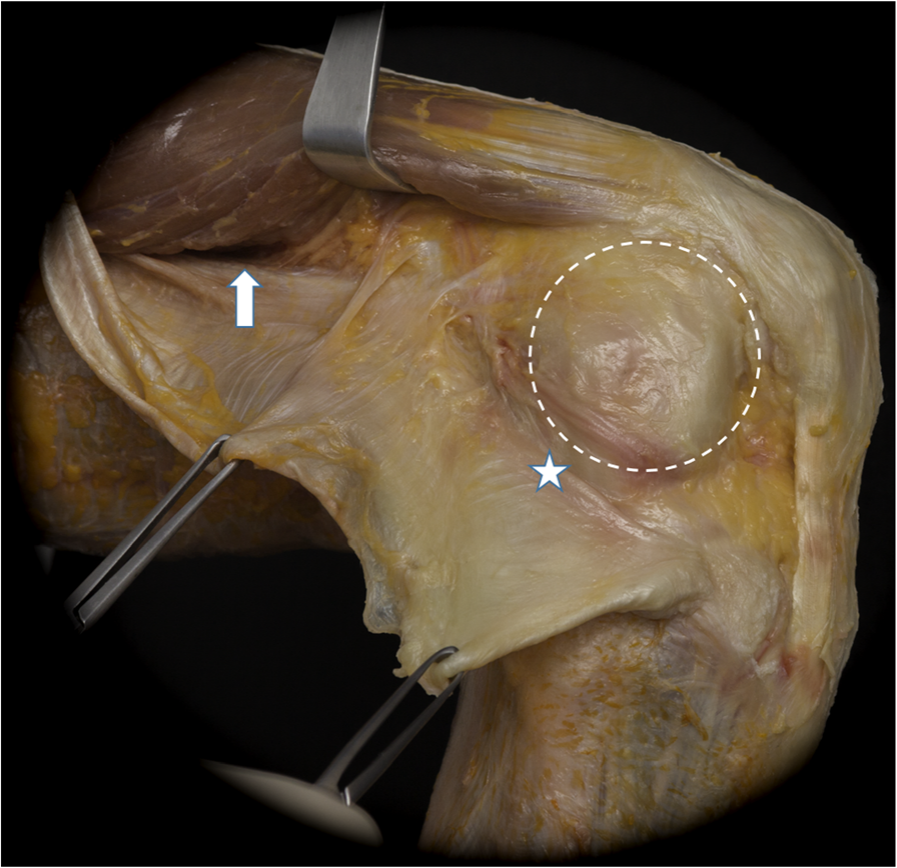
An anatomic dissection of the lateral aspect of a right knee, with the iliotibial band (ITB) cut and reflected posteriorly (within clamps). The Kaplan fibers (arrow) can be seen running from the superficial ITB, which play a role in rotatory knee stability. The posterior fibers of the ITB (star) blend with the capsulo-osseous layer and the deep ITB to insert on Gerdy’s tubercle. The lateral knee capsule (dotted circle) is also identified

An understanding of the pathoanatomy of anterolateral rotatory instability (ALRI) has been complicated by the wide variety of nomenclature used in the literature. The anterolateral stabilizing structures of the knee have been referred to by many names, including the mid-third lateral capsular layer, anterior oblique band of the fibular collateral ligament, the capsulo-osseous layer of the iliotibial band (ITB), the anterolateral ligament (ALL), the anterolateral capsule, and the anterolateral complex [28, 30] (Fig. 4).

Many structures play a role in rotatory knee stability, but similar to the nomenclature of structures, there remains controversy as to the relative role that each structure plays in maintaining rotational integrity. Early work showed that anterior cruciate ligament (ACL) incompetence is necessary for pathologic internal rotation of the tibia on the femur, as division of the anterolateral structures of the knee with an intact ACL did not produce significant tibial internal rotation on the femur [52]. In a biomechanical study of ACL-deficient and intact knees, the authors suggested that the ITB played the role of primary restraint to internal tibial rotation, particularly with greater knee flexion; however, a separate study reported that a positive pivot shift test still occurred in ACL-deficient knees with an intact ITB and a portion of the anterolateral complex known as the ALL, suggesting that these anterolateral structures are secondary stabilizers to the ACL in controlling internal tibial rotation [42, 71]. Additionally, rotatory instability, as demonstrated by the pivot shift test, is drastically improved following ACL reconstruction (ACL-R) [10]. Sectioning of the portion of the anterolateral complex described as the ALL increased internal rotation in an ACL-deficient knee by 2.7 degrees during a simulated pivot shift test, suggesting that the structures of the anterolateral knee play a secondary role in controlling tibial rotation [80]. One study showed that the fibers described as the ALL provided more significant resistance to internal rotation than the ACL at flexion angles > 30 degrees, whereas the ACL provided more significant resistance at flexion angles < 30 degrees [15, 73]. Similarly, the in situ force in the ACL during simulated pivot shift testing was significantly greater than the forces in the anterolateral corner (ALC) at low flexion angles, but significantly lower at higher degrees of knee flexion, inferring that joint position is an important factor in determining the primary component responsible for rotational integrity [4]. Moreover, the lateral meniscus and bony morphology of the distal femur have been shown to play a role in maintaining rotatory knee stability [65, 77].

Recently, efforts have been initiated to improve both the nomenclature and functional description of the anterolateral structures of the knee. The International ALC consensus group met in 2017. They concluded that the ALL is a structure with considerably variable gross morphology between individuals that resides in the anterolateral capsule, and that the ACL is the primary resistance to rotation at near extension, with secondary stabilization provided by the ITB with Kaplan fibers, lateral meniscus, ALL and anterolateral capsule [26].

#### Diagnosis

Rotatory knee instability in the ACL-deficient knee is an abnormal, complex three-dimensional motion comprised of translation and rotation along a helical axis [11, 16]. When discussing rotatory knee instability, distinction must be made between axial rotation laxity envelope, coupled rotation, and dynamic laxity [6, 16, 69, 81]. Axial rotation laxity envelope describes the maximum internal and external tibial rotation under a defined load [16, 69]. Coupled rotation refers to the obligatory internal tibial rotation that occurs during anterior tibial translation when an anterior tibial load is applied [16, 81]. The dynamic laxity of the knee, which is the tibial rotation during a giving way symptom, is assessed by the pivot shift test.

ALRI can be evaluated by manual testing consisting mainly of the pivot shift test, imaging modalities including radiographs or magnetic resonance imaging (MRI), static or dynamic measurement, navigation with dynamic radio-stereometry, stereo-dynamic fluoroscopy, opto-electronic measurement, electromagnetic measurement, and by accelerometers [16]. The pivot shift test is the only dynamic and most specific clinical test for ACL injury, as well as the most representative of knee dysfunction and predictive of patient outcome [5, 16, 32, 49]. It has been argued that dynamic radiographs have only limited significance in the evaluation of rotatory knee instability [16]. By using the Porto-Knee Testing Device (PKTD®, Soplast, Valongo, Portugal) that applies a specified anterior load and internal rotation torque to the knee, the dynamic MRI can observe rotatory knee instability with a differential cut-off value of 3.5 mm between the medial and lateral tibial plateau [16, 21]. A recent MRI study showed that even static anterior subluxation of the lateral tibial plateau of 3.0 mm or greater was associated with high-grade rotatory knee instability [51]. Besides the above mentioned imaging modalities, several systems of static measurement of rotational knee instability have been described in the last two decades [9, 56, 62, 64, 72, 84]. All static, and therefore passive, measurement methods are similar in that a special device applies a rotational torque to the lower leg while the angle of rotation is documented at defined knee flexion angles [16]. While these methods are well validated, straightforward applications, they do exhibit some limitations including possible motion between the leg and the device, the passive nature of constraints, and the requirement to measure the complete range of rotation [16]. However, various studies have postulated that the static measurements do not sufficiently describe the complex nature of rotatory knee instability [7, 33].

The pivot shift test is the most specific clinical test for ACL injury, and works by assessing kinematic dysfunction of the ACL-deficient knee during simulation of a rotatory knee injury mechanism [5, 32, 40, 87]. The pivot shift test is divided into two phases, an anterior subluxation of the lateral tibia plateau and its spontaneous reduction [25, 32]. Many studies have expressed concern regarding obtaining objective and quantitatively reliable measurements due to the variability of test application amongst different physicians [32, 70]. In order to address this concern, a standardized procedure of the pivot shift test, based on a prior published technique, was introduced at the Panther Global Summit in Pittsburgh, USA, in August 2012 [25, 32]. Another study reported that measuring the anterior translation of the lateral tibial plateau rather than global rotation could provide a convenient and reliable evaluation of the pivot shift test corresponding to a clinical grading scale [3, 16, 32]. A quantitative evaluation of the pivot shift test can be achieved with the assistance of different navigation systems using dynamic radio-stereometry, stereo-dynamic fluoroscopy, opto-electronic measurement, or electromagnetic measurement [7, 12, 14, 16, 20, 34, 39, 44, 45, 54, 74]. The disadvantages of the navigated measurement methods are their limited availability, as they cannot be used outside the operating room, are invasive, and are expensive, making them impractical in the clinical setting [16, 32]. Other techniques to quantify the pivot shift test measure the acceleration of the tibia on the femur during the pivot shift test with accelerometers or gyroscope sensors [8, 43, 53, 55, 59]. Recently, a study demonstrated a simple, reliable, and affordable quantitative evaluation of the lateral pivot shift test by a video-based image analysis measurement using the iPad [32].

#### Treatment

Surgical fixation to address pathologic anterolateral knee rotation with extra-articular tenodesis (LET) procedures has been present for decades. In the 1970’s, surgeons treated ACL-deficient knees with various LET procedures, without concomitant ACL-R, until two landmark studies illustrated that LET grafts merely provided temporary stability with poor long-term outcomes [41, 82, 94]. Due to these poor long term outcomes, surgeons addressed ACL-deficient knees with intra-articular ACL-R alone. As the years passed, various studies demonstrated no difference in functional outcomes between ACL-R and ACL-R with concomitant LET [1, 91]. Recently, however, as ACL-R failures continue to occur and surgical technique improves, renewed interest in LET procedures has arisen in order to improve rotatory control of the knee [18]. Numerous techniques have been described, performed in conjunction with ACLR, a few of which are briefly detailed below in the surgical technique section.

#### Surgical technique

**Fig. 5:**
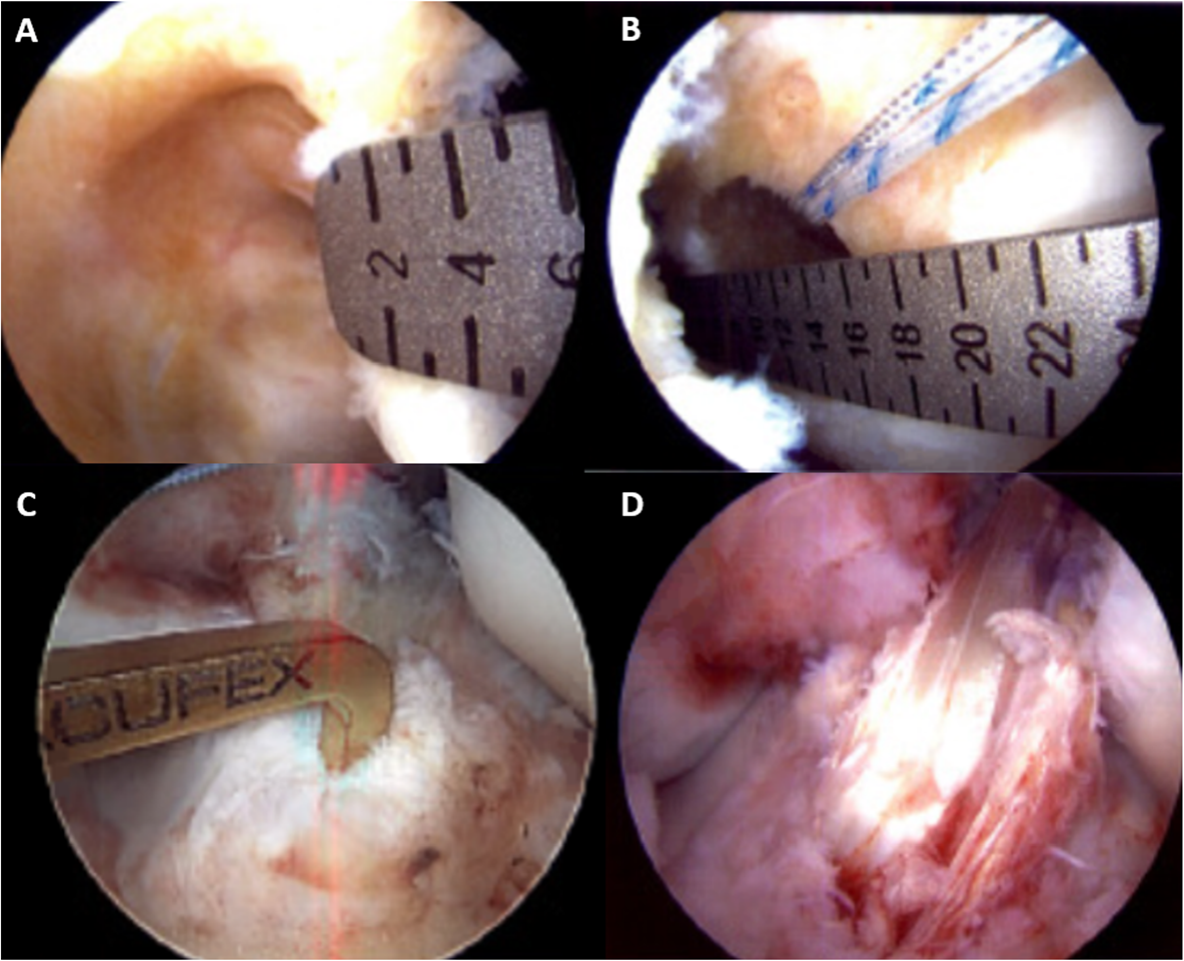
Anatomic anterior cruciate ligament (ACL) reconstruction on a left knee. **a** demonstrates 3-4 mm of posterior wall remaining after reaming the femoral tunnel, viewed from the anteromedial portal. **b** shows the anatomic position of the femoral tunnel viewed from the anteromedial portal, placed within the femoral footprint on the posterior aspect of the condyle. **c**, viewed from the anterolateral portal, demonstrates the tip aimer placed in the center of the tibia footprint. **d** demonstrates the final anatomic ACL reconstruction

Before consideration of LET procedures, a well-done anatomic ACL-R must be performed, as described previously [2, 24]. This includes placing the graft in the center of the anatomic footprints of the ACL on the tibia and femur. (Fig. 5) Once the decision is made to proceed with LET, a 5 cm incision is made on the lateral side of the knee over the distal ITB, with sharp dissection through skin and subcutaneous tissue. The ITB is identified and harvested as described below.

##### Lemaire [50]

Lemaire described the first LET procedure in 1967. This technique involves harvesting a strip of ITB, detaching it proximally, and then passing it deep to the LCL. It is looped through a drill tunnel at the origin of the lateral gastrocnemius muscle, then passed deep to the LCL and sutured to itself at Gerdy’s tubercle. The graft is tensioned with the knee in external rotation and 30 degrees of flexion.

##### Modified Lemaire [95]

A 1 cm strip of ITB is harvested and detached proximally. The graft is passed deep to the LCL and attached to the superolateral femur. The site is first prepared by decorticating it with a periosteal elevator. A small staple is used to attach the graft to the prepared insertion site, with the knee in 60 degrees of flexion and neutral rotation.

##### MacIntosh et al. [58]

This technique, detailed in 1976, involves harvesting a strip of ITB, detaching it proximally, and tunneling it deep to the LCL. An osteoperiosteal flap is then developed posterior to the origin of the LCL, under which the strip is passed. The flap is then reattached, creating a sling over the top of the iliotibial strip. The strip is tunneled through the lateral intermuscular septum, and then passed distally, deep to the LCL, and sutured to itself at Gerdy’s tubercle. The graft is tensioned with the knee in external rotation and at 90 degrees of flexion.

##### Losee et al. [57]

First described in 1978, this surgical procedure, termed the “sling and reef operation”, entails harvesting a strip of iliotibial tract and detaching it proximally. The strip is then passed deep to the LCL, through a drill tunnel starting at the superior aspect of the insertion of the lateral gastrocnemius muscle and ending at the LCL origin, and secured at Gerdy’s tubercle. The graft is tensioned with the knee in external rotation and flexed to 30 degrees.

##### Marcacci et al. [61]

This technique, described in 1998, combines ACL-R with an LET, for a combined intra-articular and extra-articular reconstruction. The semitendinosis and gracilis tendons are harvested and detached proximally, while maintaining their distal attachment. A standard ACL tibial tunnel is created, through which the graft is passed. The intra-articular graft is then passed through the femoral notch, over the top of the femoral condyle, deep to the LCL, and finally attached to Gerdy’s tubercle. The graft is tensioned with the knee in external rotation and flexed to 30 degrees.

##### Author’s technique (Fig. 6)

The authors prefer to perform a modification of the Lemaire technique whenever the patient has high grade rotatory instability, persistent intraoperative rotational instability after anatomic ACL-R, and when addressing of concomitant pathology.

**Fig. 6:**
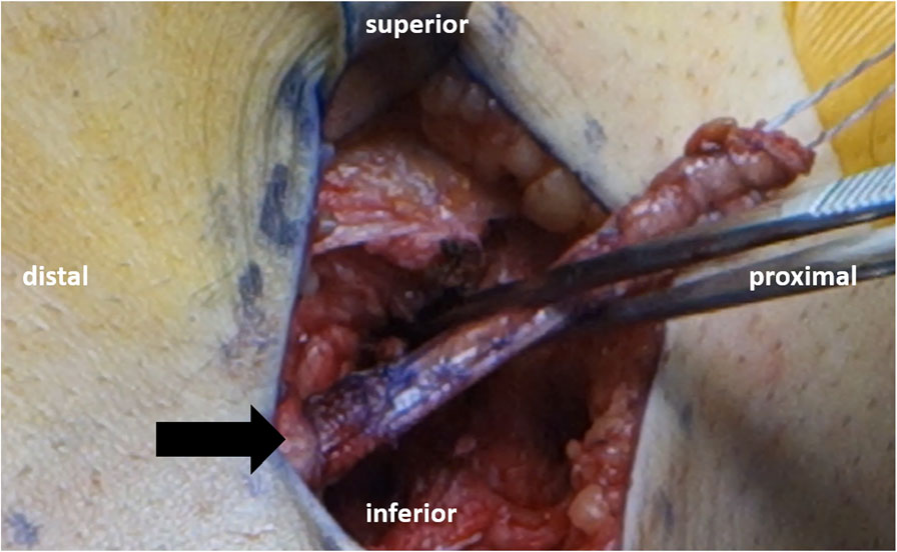
The modified Lemaire technique on a left knee. A 1 cm × 8 cm strip of iliotibial band is harvested and detached proximally. The graft is passed deep to the LCL (black arrow) and attached superolaterally to the distal femur at Lemaire’s point with a staple (forceps)

#### Concomitant pathology

It is imperative that the treating physician search for concomitant pathology in high grade rotatory knee laxity and revision cases. Bony morphology on the tibia and femur have been associated with an increased risk of ACL injury and rotatory knee laxity. An increased posterior tibial slope was found to predict high-grade rotatory knee laxity, while a smaller medial tibial depth and increased lateral tibial plateau slope have been associated with increased risk of ACL injuries [29, 78, 79]. Distal femoral characteristics, such as an increased posterior femoral condylar depth, a decreased notch width and notch width index have been associated with risk of ACL injury and persistent instability after ACL injury [38, 77, 93]. Meniscus tears, especially root tears, can cause increased rotatory knee laxity in an ACL-deficient knee [35, 85, 89]. These injuries, especially root tears, can easily be missed on preoperative MRI (Fig. 2). If these characteristics are identified preoperatively, consideration for LET in conjunction with ACL-R may be warranted, as well as osteotomies in the revision setting to address the bony pathology.

#### Outcomes

As techniques have evolved and our knowledge of the knee improved, a trend to include an LET has emerged. Initially, various authors concluded that LET over constrains the knee and results in poor long-term outcomes [68, 83]. However, recent studies have found contradictory results. One study demonstrated the addition of LET to ACL-R can be an effective procedure, and showed minimal complications at two-year follow-up [90]. Another study reported only 2 (out of 54) patients had greater than 5 mm side-to-side difference in anterior-posterior laxity at long-term follow, with 90% of the patients having good or excellent IKDC scores [60]. A recent long-term follow-up study demonstrated the addition of LET to ACL-R had improved knee stability with no increased risk of osteoarthritis, and a decreased rate of ACL failure [22]. Various studies have shown the addition of LET to an anatomic ACL-R decreased rotational knee laxity with no increased risk of osteoarthritis [22, 31, 60, 76, 89]. Most recently, a multicenter, randomized clinical trial (STABILITY I) compared anatomic ACL-R with hamstring autograft with combined ACL-R and LET, utilizing a Modified Lemaire technique. This study specifically assessed high risk patients, including those that had two of the three following criteria: generalized laxity, returning to high risk/pivoting sports, and grade 2 pivot shift or greater. The authors concluded that ACL-R with LET in a select group of young patients significantly reduces graft failure and persistent anterolateral rotatory knee laxity at 2 years post operatively [27]. Currently, there is an ongoing multicenter, randomized control trial (STABILITY II) comparing ACL-R with quadriceps tendon and bone-patellar tendon-bone autografts with and without the addition of LET.

## Conclusion

Rotatory knee instability is a complex diagnosis requiring prompt identification and appropriate surgical intervention. Various clinical and radiographic tools are available for the treating surgeon to diagnose this condition. Importantly, to address both medial and lateral rotatory knee instability patterns, the surgeon should address concomitant pathology, such as meniscus, root, or collateral ligament injury. In addition, especially in complex revision scenarios the bony morphology should be considered. The essence of reducing rotatory knee instability begins and ends with a well-done, anatomic ACL reconstruction, which may be performed with consideration of LET in a select group of patients.

## Data Availability

Not applicable

## Abbreviations

ACL: Anterior cruciate ligament
ACL-R: Anterior cruciate ligament reconstruction
ALC: Anterolateral corner
ALL: Anterolateral ligament
ALRI: Anterolateral rotatory instability
AMRI: Anteromedial rotatory instability
ITB: Iliotibial band
LCL: Lateral collateral ligament
LET: Lateral extra-articular tenodesis
MCL: Medial collateral ligament
MRI: Magnetic resonance imaging
PCL: Posterior cruciate ligament
PLC: Posterolateral corner
PLRI: Posterolateral rotatory instability
PMC: Posteromedial corner
POL: Posterior oblique ligament
